# State Recognition of Multi-Nozzle Electrospinning Based on Image Processing

**DOI:** 10.3390/mi14030529

**Published:** 2023-02-24

**Authors:** Weiqi Gao, Jiaxin Jiang, Xiang Wang, Wenwang Li, Gaofeng Zheng

**Affiliations:** 1 Department of Instrumental and Electrical Engineering, Xiamen University, Xiamen 361102, China; 2 Shenzhen Research Institute of Xiamen University, Shenzhen 518000, China; 3 School of Mechanical and Automotive Engineering, Xiamen University of Technology, Xiamen 361024, China

**Keywords:** multi-nozzle electrospinning, open-cv image processing, jet-state identification

## Abstract

The online monitoring of a multi-jet electrospinning process is critical to the achievement of stable mass electrospinning for industrial applications. In this study, the construction of an ejection state recognition system of a multi-jet electrospinning process based on image processing is reported. The ejection behaviors regarding multi-nozzle electrospinning were recorded by CMOS industrial cameras in real time. The characteristic information regarding the multi-jet cone tip was obtained by processing the images regarding Roberts operator edge detection, Hough transform line detection, and mask histogram analysis. The jet anomalies of the hanging droplets in the nozzle outlet area could be obtained and identified by the vision. The identification accuracy towards the target hanging droplets was more than 85%. This work reports the intelligent control of large-scale multi-nozzle electrospinning equipment.

## 1. Introduction

Electrospinning is one of the main methods of preparing ultrafine nanofibrous materials, which have broad applicational prospects in the fields of air filtration [1], polluted water purification [2], the development of medical dressings [3], active food packaging [4], electromagnetic interference shielding [5], and so on. Particularly, in the case of increasingly serious global water pollution, multifunctional oil–water separation nanofiber membranes are used to efficiently purify water resources [6,7,8]. The use of multi-nozzle electrospinning technology is one of the main methods employed to achieve mass electrospinning in industrial applications. Different process parameters [9], such as the spinning voltage and solution concentration, have a great influence on the formation of jets during the stretching of polymer solutions [10,11,12]. Abnormal multi-jet ejection behavior is the principal element impacting the quality and efficiency of nanofiber membrane formation. Therefore, the online monitoring of multi-nozzle electrospinning and the automatic recognition of jet anomalies have become core problems in the mass industrial electrospinning domain. 

Electrospinning utilizes a high-voltage electric field to induce a charged jet and thus produces nanofibers from a polymer solution, and typsof this technique include single-nozzle or multi-nozzle electrospinning and needleless electrospinning. At present, stable multi-nozzle spinnerets are available; for example, Jiang [13] et al. designed a new spinneret structure in which the nozzles are arranged in an arc array and simulated and adjusted the electrical field distribution among the nozzles by using the Ansys software to achieve a relatively uniform electrical field. In addition, the following work described in this paper has been carried out based on the spinneret mentioned above. Clearly, online multi-jet detection and the automatic recognition of multi-jet anomalies is necessary to guarantee the industrial generation of mass electrospinning. However, there are some difficulties that must be solved, such as those regarding strong interferences, small size, and high speed in the electrospinning process. The adjustment of the electrospinning current that stems from charge migration is one of approaches used to monitor ejection behaviors. Samatham [14] et al. established a microcurrent test system and explored a method to monitor electrospinning states through current changes. Kim [9] et al. studied the influence of the electrospinning current on the morphology of nanofibers and optimized the process parameters to increase the uniformity of electrospun nanofibers. Zheng [15] et al. identified and analyzed the various ejection modes of an Electrohydrodynamic Direct-Write (EDW) system by introducing image recognition and microcurrent detection. Nevertheless, because the electrospinning current has the characteristics of small values, strong interferences, and a low signal-to-noise ratio, it is difficult to determine multi-nozzle jet states online. Alternatively, visual detection is another great way to realize online monitoring [16], which offers the advantages of high efficiency and intuitiveness. Kadomae [17] et al. used industrial cameras to collect images of the liquid’s shape at the cone tip to study the printing behaviors via monitoring the jet mode. Choi [18] et al. sprayed zeolite dispersion at a higher position than the Taylor cone and captured dozens of images of the jet with some particles by using a high-speed camera to successfully measure the jet velocities. Li [19] et al. used a high-speed camera to record the jet movement in three different melt electrospinning systems to study the influence of the electric field distribution on the jets’ movement. Mieszczanek [20] et al. monitored and analyzed the jet angle and area of the first Taylor cone with a high-resolution camera in melt electrowriting to further detect and correct the degree of fiber pulsing. Li [21] et al. used a high-speed camera to capture the images of the Taylor cone and jet motion, proving that different PVP concentrations in the core and shell could lead to different core and shell thicknesses as well as various jet modes. The above research mainly focuses on the Taylor cone area and jet modes of the single-nozzle-electrospinning process, which is quite different from the monitoring requirements of multi-nozzle electrospinning. In addition, the latter is required to meet the demand for a larger recognition range, a greater information volume, and the lower information reliability of multi-jet state images. Meanwhile, the purpose of this research work is not to accurately identify the jet ejection behavior pattern, but to identify whether the jet in the nozzle’s exit area is stable. 

In this paper, aiming to develop a convenient detection and identification method for state recognition applied to multi-nozzle electrospinning, an online multi-jet state identification system was established based on Python and Open-cv, which can identify the jet anomalies of the nozzle outlet area and display warning signs. If there is a hanging drop instead of a cone jet at the nozzle outlet, it is judged as abnormal ejection. This work promotes the development ofan intelligent control technology towards the realization of mass electrospinning for the efficient production of nanofilms.

## 2. Materials and Methods

Polyethylene (PEO, *M_ω_* = 3 × 10^5^ g·mol^−1^, Changchun Dadi Fine Chemical Co., Ltd., Changchun, China) was selected as the analyzed material. The solvent was deionized water/ethanol (*v:v* = 3:1) mixed solvent. The mass concentration of PEO solution is 10 ωt%. 

**Figure 1:**
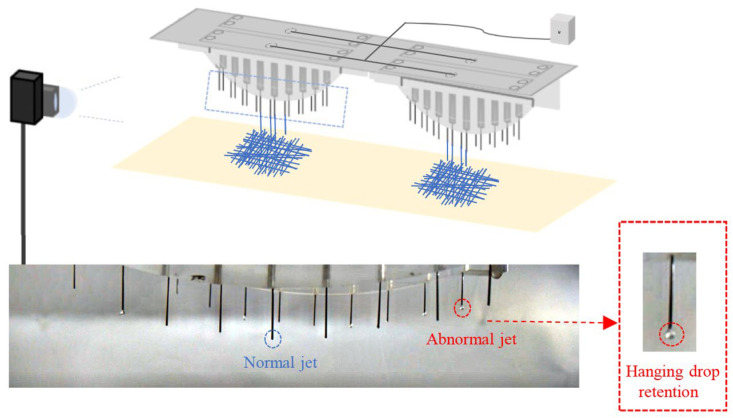
Schematic diagram of multi-nozzle electrospinning equipment.

A schematic of a multi-jet-state-monitoring system is shown in Figure 1; notably, this work only concerns the nozzle outlet area. A precision syringe pump (Pump 11 Pico Plus Elite, Harvard Apparatus America, Dover, MA, USA) was used to deliver solution continuously and quantitatively to the spinneret. A DC high-voltage power supply (DW-SA403-1ACE5, Dongwen high-voltage power source Ltd., Tianjin, China) was used to generate a strong electric field between the spinneret and the collector. The anode of the DC high-voltage power supply was connected to the nozzle of the spinneret, and the cathode was connected to the collector. A complementary metal-oxide-semiconductor (CMOS) camera (MV-CS050-10GC, Hangzhou Hikvision Digital Technology Ltd., Hangzhou, China) was used to observe and record the ejection state of the multi-nozzle electrospinning system in the outlet area.

A mass multi-nozzle electrospinning device was equipped with multiple CMOS industrial cameras for real-time recording. As shown in Figure 1, two multi-jet spinnerets could be regarded as one group, which was recorded by the same camera. Based on the recorded images of the electrospinning process, the jet anomalies of the nozzle outlet area, namely, hanging droplets, have been identified to ensure that the device can produce stable and uniform nanofibers in the industry.

## 3. Multi-Jet Image Processing

**Figure 2:**
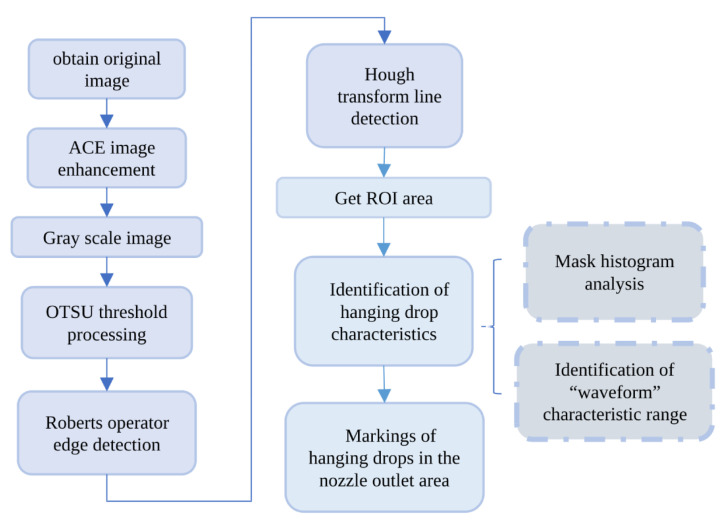
A processing flow chart of multi-jet state identification system.

The monitoring of the multi-jet electrospinning process has been transformed into obtaining real-time images recorded by a CMOS camera. Based on image-processing technology, the Regions of Interest (ROI) of the images were established, and the hanging drops of the cone tips were recognized. The processing flow of the multi-jet electrospinning imagesis shown in Figure 2.

### 3.1. Image Preprocessing

**Figure 3:**
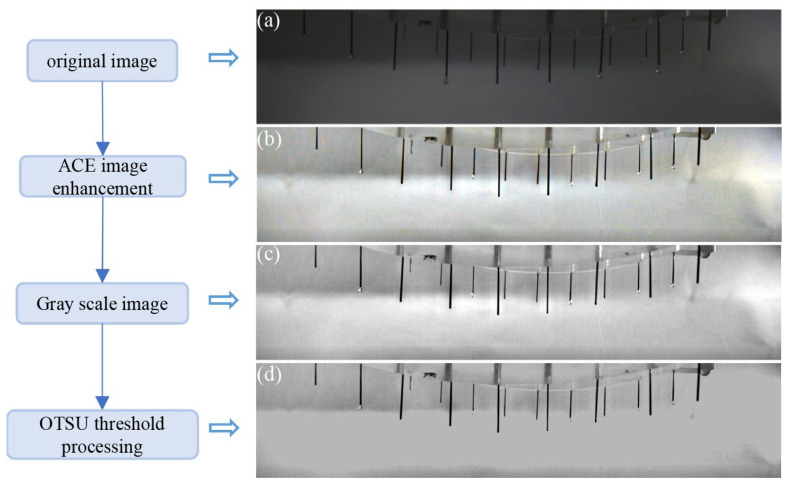
The process diagram of image preprocessing: (**a**) Original image; (**b**) the result after ACE image enhancement; (**c**) Gray scale image; (**d**) the image after OSTU threshold processing.

There was little difference in the grey values between the target objects and the background. In order to obtain more accurate and reliable results, the initial images needed to be initially adjusted. The preprocessing stage and its results are shown in Figure 3.

#### 3.1.1. ACE Algorithm

Automatic color enhancement (ACE) is based on the theory of the Retinex algorithm [22,23], and it corrects the final pixel value by combining the brightness degree and distance between the target pixels and the surrounding pixels in order to achieve good image contrast adjustment.

The ACE algorithm consists of two steps: (1) the adjustment of the color and space of the image to complete the correction of color difference; (2) the dynamic expansion of the corrected image [24]. Its calculation formula is as follows:

(1) $$Y=\frac{\Sigma^{\;}{(\;g(\;I(x}_{0})\;-\;I(x)\;)\;\cdot {\;\omega (x}_{0\;},\;x)\;)}{\Sigma^{\;}{(\;\omega (x}_{0}\;,\;x)\;)}$$

In this formula, *ω* is the weight coefficient, *g* is the relative contrast adjustment parameter *g*(*x*) = max (min (*ax*, 1.0), −1.0), and *a* is the control parameter.

In this paper, the ACE algorithm was used to process the initial image. Figure 3b depicts the result after image enhancement. While maintaining the authenticity of the image, the light and dark degrees of the image were greatly improved, thereby strengthening the contrast between the target object and the background pixel.

#### 3.1.2. Threshold Processing

The maximum inter-class variance method proposed by the Japanese scholar Nobuyuki Otsu (OTSU) was used to select a threshold at which to divide the image into foreground and background parts [25] and then calculate the inter-class variance of the two parts. The larger the inter-class variance, the more appropriate the selected threshold. The definition and the calculation formula of interclass variance are as follows:

(2) $$\sigma^{2}{=P}_{1}{\;(m}_{1}\;{-\;m}_{G}{)}^{2}{+P}_{2}{\;(m}_{2\;}{-\;m}_{G}{)}^{2}$$

In this formula, σ is the inter-class variance; $P_{1}{,\;P}_{2}$ are the probability of pixels appearing as foreground and background regions, respectively; and $m_{1}{,\;m}_{2}{,\;m}_{G}$ are the foreground, background, and global average gray values, respectively.

The three-channel image processed by the ACE algorithm was grayed to convert it into a single-channel image, as shown in Figure 3c. In addition, OTSU threshold processing was used to automatically select an appropriate threshold to cope with various gray background values, as shown in Figure 3d.

### 3.2. Hough Transform Edge Detection

**Figure 4:**
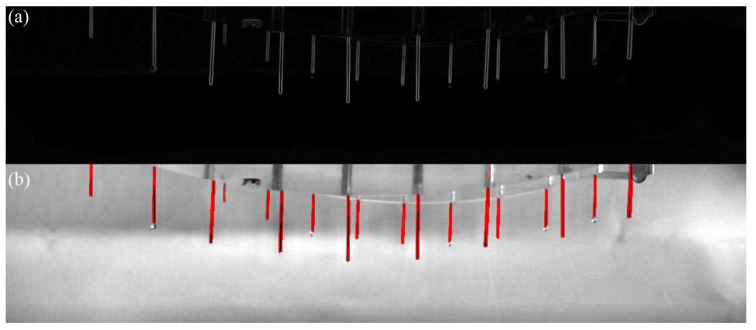
Edge detection of spinneret cone wall: (**a**) Roberts operator contour detection; (**b**) Hough transform line detection.

The Roberts operator is a gradient algorithm based on cross difference, which detects edge lines by local difference calculation. The algorithm has a remarkable effect on image edge enhancement of ±45°. In this paper, the Roberts operator was used to detect the edge of the spinneret cone wall, and the result is shown in Figure 4a. 

On this basis, the contour information was obtained via Hough transform, which was widely used for curve detection in image processing. In addition, in this paper, the cumulative probability Hough transform was adopted. Its basic principle is to transform the pixels of an image from the Cartesian coordinate system to the polar Hough space system, and then accumulate and randomly extract the fitting points until the number of fitting lines reaches the threshold. The polar transformation formula of the Hough transform is as follows:

(3) r=x⋅cosθ+ysinθ

In the formula, *r* is the distance from the origin to the nearest point on a straight line, and θ is the angle between the *x* axis and the straight line.

Before initiating line detection, in order to improve the system’s recognition accuracy, binarization processing and morphological operation were used to remove marginal fine noise. Then, the target line segments were selected according to the vertical slope and an appropriate range of length to exclude the interfering curves. The detection results are shown by the red lines in Figure 4b. Furthermore, the coordinates of the target line segment’s endpoints were obtained. The appropriate endpoint coordinates were selected as the center coordinates of the circular area to establish the ROI. In addition, the hanging drop feature was extracted and analyzed in this area.

## 4. Results and Discussion

**Figure 5:**
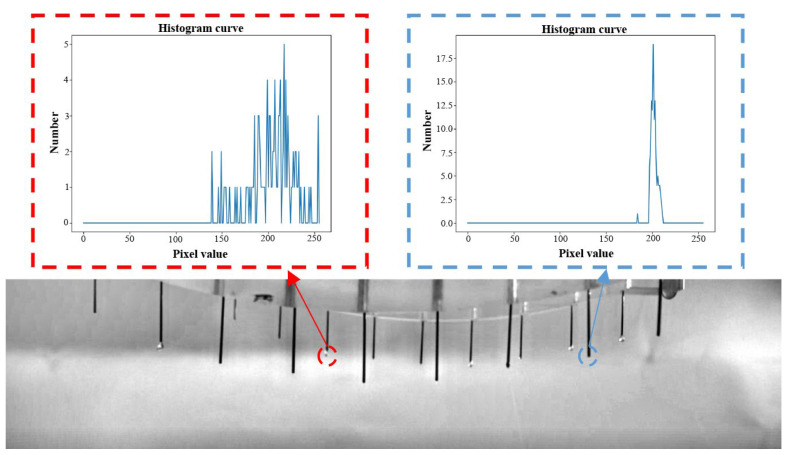
The mask histogram analysis.

In the nozzle outlet areas of the images, the hanging drops at the spinneret cone tips could be found. Due to the refraction and scattering of light, there are obvious pixel changes in this area, which have an approximately waveform-like shape. The mask histogram analysis of the ROI is shown in Figure 5. In this region with its hanging drops, the pixel changed obviously, and the distribution was scattered. Conversely, when there were no hanging drops in the cone tip area, the background did not fluctuate, and the pixel distribution in the histogram was concentrated.

In this paper, two methods for the detection of hanging drops at the nozzle outlet area were adopted. Firstly, the mean square deviation threshold was set to screen and identify the hanging droplets. The mean square deviation can accurately describe the distribution of a histogram. The larger the mean square deviation, the more scattered the pixel distribution of the histogram. Secondly, the coordinate distribution range of the cone tip was compared with the pixel fluctuation range of the ROI. If the pixel fluctuation range in the ROI is greater than the distribution range of the cone tip coordinate, it was surmised that there was a hanging droplet. Combining the results of the two detection methods, the warning indications were defined. 

**Figure 6:**
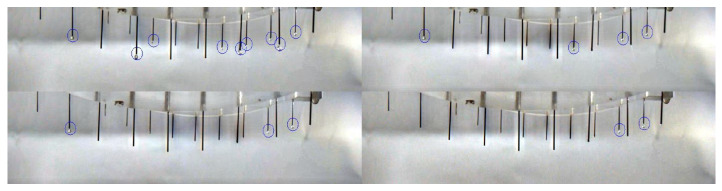
Multi-jet state identification in different periods.

Multiple groups of video-framing outputs were selected to verify the results. The accurate identification rate of hanging droplets at the multi-jet cone tip reached 85%. The specific verification data are shown in Table 1, including the total frames, the total number of multi-jet cone tips, the number of correct frames and error frames, and the accurate identification rate. In addition, as shown in Figure 6, the identification results of different time states in the same group have been displayed. The warning marks of hanging drops in the nozzle outlet area were indicated.

## 5. Conclusions

Based on Python and Open-cv, an online multi-jet state identification system was built, which realized the monitoring of the multi-jet ejection state and the recognition of hanging droplets in the nozzle outlet area. CMOS industrial cameras were used to obtain the original images. The ACE algorithm and OTSU threshold processing effectively solved the problem wherein the overall background of the original image was dim and the difference in the grey values between the target pixel and the background pixel was small. After image preprocessing, the Roberts operator edge detection and Hough transform line detection adaptively obtained the endpoint coordinates of the multi-jet cone tips and established the ROI area. According to the regional pixel fluctuation of the hanging drops in the ROI, histogram analysis and pixel fluctuation detection were carried out to effectively identify the jet anomalies of hanging droplets in the nozzle outlet area. The feedback recognition results of image processing were consistent with the actual situation, which was conducive to achieving the intelligent control of large multi-nozzle electrospinning equipment.

## Figures and Tables

**Table 1:** Multiple sets of multi-jet state recognition data.

Number	Total Frames	Total Number of Cone Tips	Correct/Frame	Error/Frame	Accuracy Rate
1	193	3474	165	28	85.5%
2	306	5508	265	41	86.6%
3	682	12,276	597	85	87.5%

## Data Availability

The data presented in this study are available on request from the corresponding author.
